# Salmeterol/fluticasone stable-dose treatment compared with formoterol/budesonide adjustable maintenance dosing: impact on health-related quality of life

**DOI:** 10.1186/1465-9921-8-46

**Published:** 2007-07-04

**Authors:** David B Price, Angela E Williams, Sally Yoxall

**Affiliations:** 1 Department of General Practice and Primary Care, University of Aberdeen, Foresterhill Health Centre, Westburn Road, Aberdeen AB25 2AY, UK; 2 Research and Development, GlaxoSmithKline, Greenford Road, Greenford, Middlesex UB6 0HE, UK

## Abstract

**Background:**

Improving patients' health-related quality of life (HRQoL) is recognized as a fundamental part of asthma management. The aims of this study were to evaluate the long-term efficacy (including symptom-free days and exacerbations) and impact on HRQoL of a stable-dose regimen of salmeterol/fluticasone propionate (SAL/FP) and an adjustable maintenance dosing (AMD) regimen of formoterol/budesonide (FOR/BUD) where treatment is adjusted based on symptoms [SAM40056].

**Methods:**

A total of 688 outpatients with asthma receiving regular low-dose inhaled corticosteroids (ICS) plus a long-acting β_2_-agonist, or medium dose ICS alone participated in this randomized, double-blind, double-dummy, parallel-group, 1-year trial, which was conducted in 91 centers in 15 countries. Patients were randomized to receive 1 inhalation of SAL/FP 50/250 μg BID or 2 inhalations of FOR/BUD 6/200 μg BID during Weeks 1–4. For Weeks 5–52, patients meeting strict continuation criteria for stable asthma at Week 4 received AMD with FOR/BUD or stable-dose SAL/FP.

**Results:**

The percentage of symptom-free days was significantly greater (58.8% vs 52.1%; p = 0.034) and the annual exacerbation rate was significantly lower (47%; p = 0.008) with stable-dose SAL/FP compared with FOR/BUD AMD. A total of 568 patients completed the Asthma Quality of Life Questionnaire (AQLQ) at least once during the study. The mean change from baseline in AQLQ overall score was numerically greater with SAL/FP than FOR/BUD at week 28 and week 52, but did not reach statistical significance (p = 0.121 at Week 52). However, in a post hoc logistic regression analyses for any AQLQ improvement, significant benefits with SAL/FP were seen at both time points (p = 0.038 and p = 0.009, respectively). The minimally important difference of ≥ 0.5-point improvement in AQLQ overall score was achieved by a significantly greater number of patients receiving SAL/FP at Week 28 (68% vs 60%; p = 0.049); a trend for this difference remained at Week 52 (71% vs 65%) (p = 0.205).

**Conclusion:**

In this population of patients with persistent asthma, stable-dose SAL/FP resulted in significantly greater increases in symptom-free days, a reduction in exacerbation rates, and provided greater HRQoL benefits compared with FOR/BUD AMD.

**Trial registration:**

Clinical Trials registration number **NCT00479739**

## Background

The goals for successful management of asthma are defined as achieving and maintaining symptom control, preventing exacerbations, maintaining lung function as close to normal as possible, preventing asthma mortality and development of irreversible airflow limitation, maintaining normal activity levels and avoiding treatment-related adverse effects [1]. In practice, these goals are rarely achieved and asthma control remains poor, with many patients continuing to suffer frequent symptoms and exacerbations [2]. Asthma also has a substantial impact on the health-related quality of life (HRQoL) of patients, with the physical, emotional and social aspects of their lives often considerably impaired [1]. Indeed, in a study investigating patient-defined treatment success, 'improved ability to do normal things' was found to be one of the most important treatment goals, second only to reducing the number of exacerbations [3]. Asthma management guidelines, therefore, recognize the importance of improving patients' daily functioning and wellbeing in addition to improving objective clinical measures of asthma control (e.g. exacerbation reduction) [1]. However, recent research using factor analysis to explore the relationships between HRQoL, measured using the Asthma Quality of Life Questionnaire (AQLQ), and conventional clinical endpoints has demonstrated that HRQoL is a distinct component of asthma health status and is independent of symptom scores or lung function [4]. This supports previous findings that HRQoL measures correlate poorly with clinical parameters, including symptoms and airway caliber [5-9].

Hence, it is not only important for clinical trials to demonstrate that efficacy improvements in response to treatments are clinically meaningful in terms of traditional clinical measures such as control of symptoms and exacerbations; it is also important that they provide further insight from the patient by including an assessment of HRQoL – a patient-reported outcome – using a validated instrument such as the AQLQ. Indeed, the American Thoracic Society and the European Medicines Agency have both issued guidance relating specifically to the measurement of HRQoL [10,11], and the US Food and Drug Administration plan to develop guidance for using patient-reported outcomes during 2005 [12].

In asthma management guidelines, a combination of an inhaled corticosteroid (ICS) and a long-acting β_2_-agonist (LABA) is recommended for the treatment of patients not controlled on low or moderate doses of ICS alone [1]. There are two combinations of LABA/ICS currently available in a single device, salmeterol/fluticasone propionate (SAL/FP) and formoterol/budesonide (FOR/BUD), both of which have been demonstrated to significantly improve patients' HRQoL in one-year studies [13,14]. The adjustable maintenance dosing (AMD) regimen, which allows patients to adjust their dose of FOR/BUD according to the severity of their symptoms, has been shown to reduce asthma exacerbations compared with fixed dosing in open-label studies [15-17]. This randomized, double-blind, double-dummy trial – the CONtrol CEntred Patient Treatment (CONCEPT) – is the first study of this design to have investigated the efficacy and HRQoL effects of stable-dose SAL/FP via Diskus^® ^(trademark of GlaxoSmithKline, Ware, UK) compared with an AMD regimen of FOR/BUD via Turbuhaler^® ^consistent with the current license (trademark of AstraZeneca, Södertälje, Sweden) [18].

## Methods

### Patients

Full details of the study design and methods have been previously reported [18]. Male and female outpatients (aged 18-<70 years) with a documented clinical history of asthma and forced expiratory volume in 1 second (FEV_1_) 60–90% of predicted normal were enrolled in the study. All patients had received an ICS dose equivalent to 200–500 μg/day beclomethasone dipropionate (BDP) plus a LABA, or ICS alone at a dose equivalent to > 500–1000 μg/day BDP for at least 12 weeks before enrollment. Patients who met any of the following criteria were excluded: a lower respiratory tract infection or use of systemic corticosteroids within 1 month prior to study entry, a ≥ 10 pack-year smoking history, changes to regular asthma therapy within 12 weeks of study entry, or any significant disorder that may put the patient at risk or influence study outcomes. Inhaled cromones, leukotriene modifiers, β_2_-agonists (except salbutamol as rescue medication), xanthines, and inhaled anticholinergics were not permitted during the study.

### Study design

This was a randomized, double-blind, double-dummy, parallel-group study conducted in 91 centers in 15 countries. During the 2-week run-in period, patients who showed a total daily symptom score of ≥ 2 on at least 4 of the last 7 evaluable days were eligible for randomization to the 52-week treatment period, which comprised two phases. During the first phase (Weeks 1–4), patients received either 1 inhalation of SAL/FP 50/250 μg BID via Diskus plus 2 inhalations of placebo BID via Turbuhaler or 2 inhalations of FOR/BUD 6/200 μg BID (equivalent to 4.5/160 μg delivered dose) plus 1 inhalation of placebo BID via Diskus.

Patients were eligible to enter the second treatment phase of the study (Weeks 5–52) if they reported no night-time awakenings due to asthma and no salbutamol use on >2 days in their diary cards during the 7 days before Visit 3 (i.e. during Week 4). For patients who met these criteria, the Turbuhaler dose was reduced to 1 inhalation BID, with further reduction to 1 inhalation/day if the criteria continued to be met at subsequent visits. If the criteria were not met at later visits, patients reverted to 1 inhalation BID. Patients also received oral and written information about the AMD self-management plan. Step up of treatment was patient-initiated, whereas stepping down was initiated in consultation with the investigator. The step-up and step-down criteria are detailed in Table 1, and were based on previously published studies [15-17]. For patients with persistent symptoms remaining after 14 days of 4 Turbuhaler inhalations BID, the investigator prescribed a short course of oral corticosteroids and instructed the patient to step down to 1 inhalation BID. Stable dosing with 1 inhalation BID via Diskus was maintained throughout the 52-week, double-blind treatment period. The study was conducted in accordance with the Declaration of Helsinki and good clinical practice guidelines. The study protocol, patient information form and informed consent form were approved by the local ethics committees. All patients provided written informed consent before beginning the study.

**Table 1:** Adjustable maintenance dosing plan for active treatment or placebo administered via Turbuhaler^a ^during Weeks 5–52.

**Adjustment**	**Criteria**
Step up: from 1 or 2 inhalations/d to 4 inhalations BID (judged by the patient)	Two consecutive days or nights with: Rescue medication used ≥ 3 times during the day OR Night-time awakening due to asthma OR Morning PEF <85% of the mean of the last 7 days before Visit 3
Step down: from 4 inhalations BID to 1 inhalation BID after 7–14 days of step-up treatment (judged by the investigator)	Last 2 consecutive days or nights with: No rescue medication use OR No night-time awakening due to asthma OR Morning PEF ≥ 85% of the mean of the last 7 days before Visit 3

PEF = peak expiratory flow.

^a^Turbuhaler is a trademark of AstraZeneca, Södertälje, Sweden.

### Statistical methods and analysis

The primary efficacy variable was the percentage of symptom-free days, defined as a 24-hour period with a symptom score of 0 (recorded in patients' daily diaries). Additional parameters included the rate of exacerbations, defined as a worsening of asthma requiring hospital treatment or treatment with oral corticosteroids (based on investigator opinion or ≥ 2 consecutive days with morning peak expiratory flow (PEF) ≤ 70% of the mean of the last 7 days of Weeks 1–4).

Efficacy analyses were based on the Intent To Treat (ITT) population (patients who took ≥ 1 dose of study medication and had ≥ 1 post-randomization diary assessment). The percentage of symptom-free days was compared between treatment groups using the van Elteren extension to the Wilcoxon rank-sum test, stratified by country grouping. The exacerbation rate was calculated using a maximum likelihood-based analysis assuming the negative binomial distribution, with time on treatment as an offset variable, and the model included adjustments for treatment, sex, country grouping, and age.

The effects of the different treatments on HRQoL were evaluated using the AQLQ at Week 0 (baseline) and Weeks 28 and 52. A within-subject change of 0.5 points was considered the minimal important difference (MID), with a change of ≤ 1 defined as 'minimal' change, and >1 as 'moderate' change [19,20]. Mean change from baseline and the distribution of change in AQLQ overall scores were analyzed using analysis of covariance. There was an *a priori *intent to analyze the proportions of patients achieving MID improvement or deterioration, which was further developed with a post hoc analysis using logistic regression (adjusted for age, sex, country, and baseline AQLQ score), and the overall change in AQLQ at Weeks 28 and 52 investigated in a post-hoc proportional odds regression analysis. A post-hoc descriptive exploratory analysis investigated how many patients in each group demonstrated treatment success defined according to AQLQ changes plus the occurrence of exacerbations (success = AQLQ change of ≥ 0.5 and no exacerbations; no change = AQLQ change of >-0.5 to <0.5 and no exacerbations; failure = ≥ 1 exacerbations with any AQLQ change or AQLQ change =-0.5 with no exacerbations). For patients to be included in the post-hoc analysis they needed to have an AQLQ response at the particular visit to be analyzed (i.e. at Week 28 or Week 52).

## Results

A total of 568 patients in the ITT population (82.6%) completed the AQLQ at least once during the study; the numbers of patients who completed the AQLQ at each time point are summarized in Table 2. The baseline characteristics of this AQLQ population were similar to those of the ITT population (Table 3).

**Table 2:** Summary of AQLQ completers at each time point during the study

**Timepoint**	**SAL/FP**	**FOR/BUD**
Total ITT population	344	344
AQLQ completers		
At least once during study	280	288
Baseline	278	286
Total Week 5–52 population	295	286
AQLQ completers		
Week 28	173 (59)^†^	166 (58)^†^
Week 52	158 (54)^†^	155 (54)^†^

AQLQ = Asthma Quality of Life Questionnaire; FOR/BUD = formoterol/budesonide combination; ITT = intent to treat; SAL/FP = salmeterol/fluticasone propionate combination.

^† ^Percentage of Week 5–52 population

**Table 3:** Baseline characteristics of the ITT population and patients who completed the AQLQ at least once during the study

	**ITT population**		**AQLQ population**^a^	
**Characteristics**	**SAL/FP**	**FOR/BUD**	**SAL/FP**	**FOR/BUD**

No. of patients	344	344	280	288
Age, mean (SD), years	46 (14)	44 (14)	45 (14)	44 (14)
Sex, female no. (%)	204 (59)	216 (63)	156 (56)	178 (62)
Asthma Duration ≥ 10 years no.(%)	197 (57)	200 (58)	171 (61)	162 (56)
FEV_1_, mean (SD), L	2.53 (0.80)	2.52 (0.70)	2.57 (0.83)	2.49 (0.68)
FEV_1_, mean (SD), % predicted	82 (21)	81 (13)	82 (23)	80 (12)
Daily asthma symptom score, mean (SD)	1.9 (0.6)	1.9 (0.5)	2.0 (0.6)	1.9 (0.6)
AQLQ overall score, mean (SD)			4.8 (1.0)	4.8 (0.9)

AQLQ = Asthma Quality of Life Questionnaire; FOR/BUD = formoterol/budesonide combination; ITT = intent to treat; SAL/FP = salmeterol/fluticasone propionate combination; SD = standard deviation.

^a ^ITT patients who completed at least one AQLQ questionnaire.

### Efficacy

Stable dosing with SAL/FP resulted in a significantly higher percentage of symptom-free days compared with AMD with FOR/BUD over the whole 52-week treatment period (58.8% vs 52.1%; p = 0.034). Similarly, the percentage of symptom-free days was significantly higher with SAL/FP during Weeks 5–52 (73.8% vs 64.9%; p = 0.030). Furthermore, the adjusted annual mean exacerbation rate was 47% lower in the SAL/FP group compared with the FOR/BUD group (0.18 vs 0.33; adjusted treatment rate ratio 0.53 [95% CI: 0.34–0.85]; p = 0.008). Over the 52-week the mean daily ICS exposure was 463 (81) μg FP, in the SAL/FP group, and 480 (238) μg FP in the FOR/BUD group. Efficacy results have been reported in detail elsewhere [18].

### Health-related quality of life

The mean AQLQ overall score at baseline was similar in the two groups (3). The mean change from baseline in AQLQ overall score was greater with stable-dose SAL/FP compared with AMD of FOR/BUD after both Week 28 (1.0 vs 0.8) and Week 52 (1.1 vs 0.9). Statistical significance was tested at the end of the 52-week study period and the difference was found to be not significant (p = 0.121).

The *a priori *and post-hoc regression analyses showed that any degree of improvement in AQLQ was achieved by a significantly greater proportion of patients receiving stable-dose SAL/FP compared with the AMD FOR/BUD group at both week 28 (90% vs 83%; p = 0.038) and week 52 (91% vs 81%; p = 0.009). Similarly, a proportional odds regression analysis, performed post hoc on the categories MID improvement and less than MID improvement/no change against deterioration in HRQoL, showed that the odds ratios for SAL/FP:FOR/BUD at Weeks 28 and 52 were 1.72 (95% CI: 1.08–2.73; p = 0.022) and 1.65 (95% CI: 0.99–2.75; p = 0.057) respectively. The overall proportions of patients with at least an MID improvement, a less than MID improvement/no change and deteriorating HRQoL at Week 52 are shown in Table 4.

**Table 4:** Patients achieving at least an MID improvement, a less than MID improvement/no change or with deteriorating QoL at Week 52

**Week 52**	**AQLQ Population**	
n (%)	**SAL/FP** **n = 158**	**FOR/BUD** **n = 155**

Deterioration in QoL (<0)	14 (9)	29 (19)
No Change/Improvement <MID (≥ 0 – <0.5)	32 (20)	26 (17)
At Least MID Improvement in QoL (≥ 0.5)	112(71)	100 (64)

AQLQ = Asthma Quality of Life Questionnaire; FOR/BUD = formoterol/budesonide combination ; SAL/FP = Salmeterol/fluticasone propionate combination; QoL = Quality of Life; MID = minimally important difference.

**Figure 1:**
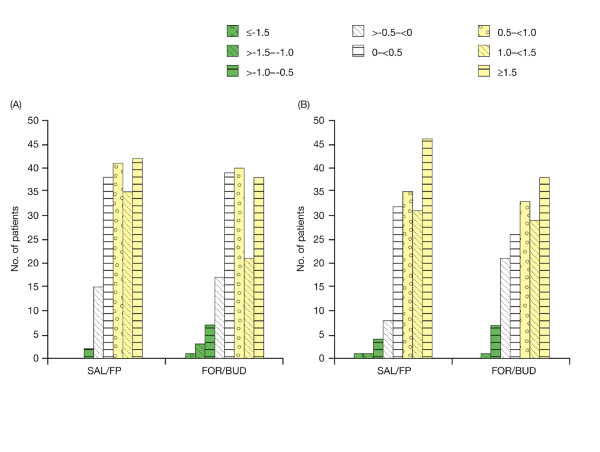
**Distribution of change in AQLQ overall score at (a) 28 weeks and (b) 52 weeks**. AQLQ = Asthma Quality of Life Questionnaire; FOR/BUD = formoterol/budesonide combination; SAL/FP = salmeterol/fluticasone propionate combination.

Looking at the distribution changes in AQLQ score at Week 28 (Figure 1a), 1% of the SAL/FP group showed deterioration in AQLQ overall score of ≥ 0.5-point, compared with 7% of the FOR/BUD group, translating into net benefits (i.e. proportion of patients improving less those deteriorating) of 67% and 53% respectively. At 52 weeks (Figure 1b), MID deterioration occurred in 4% of the SAL/FP group and 5% of the FOR/BUD group, translating into net benefits of 67% and 60%, respectively

The post hoc exploratory analysis classifying treatment success or failure based on both AQLQ changes and exacerbations showed that more patients in the SAL/FP group achieved treatment success compared with those in the FOR/BUD group (63% vs 54%), whereas fewer SAL/FP-treated patients were classed as showing no change (23% vs 26%) or failure (14% vs 21%) (Table 5).

**Table 5:** The percentage of patients achieving treatment success or failure defined according to AQLQ change and exacerbations

**Category**	**AQLQ change from baseline at Week 52**	**Exacerbation**	**SAL/FP** **n = 158** **n (%)**	**FOR/BUD** **n = 155** **n (%)**
Success	≥ 0.5	No	100 (63)	83 (53)

No change	>-0.5 – <0.5	No	36 (23)	40 (26)

Failure			22 (14)	32 (21)
	≥ 0.5	Yes	12 (7)	17 (11)
	>-0.5 – <0.5	Yes	4 (3)	7 (5)
	≤ -0.5	No	4 (3)	3 (2)
	≤ -0.5	Yes	2 (1)	5 (3)

AQLQ = Asthma Quality of Life Questionnaire; FOR/BUD = formoterol/budesonide combination; SAL/FP = salmeterol/fluticasone propionate combination.

## Discussion

This is the first study to compare the long-term effects of stable dosing with SAL/FP and an AMD regimen with FOR/BUD on the HRQoL of patients with asthma. The results show that there was improvement in both groups as indicated by the mean change from baseline. However, although greater with stable-dose SAL/FP compared with AMD FOR/BUD, this was not significant. In the post-hoc proportional odds regression analysis, the odds ratio for any improvement in AQLQ against no improvement or deterioration showed a significant benefit for a stable dosing regimen of SAL/FP over a AMD dosing regimen of FOR/BUD at week 28 while there was a trend for benefit at week 52. It is important to note that the use of a double-blind, double-dummy design minimized any potential for bias resulting from patients knowing which of the treatments was active, a particularly important consideration when both patients and investigators are involved in decisions about medication adjustments. Although the complexity of the study would be decreased with an open-label design, any control over the influence of such bias would be lost. Differences in the licenses for the two study medications may have contributed to the results seen in this analysis, in particularly the license for FOR/BUD allow patients to reduce to one puff/day. This may be more of a risk in the patients with moderate asthma included in this study. With regard to daily, ICS exposure during the 52-week study, as described in the primary paper (18), for the ITT population the mean (SD) daily ICS exposure in the SAL/FP group was 463 (81) μg FP; in the FOR/BUD group, the mean daily ICS exposure was 480 (238) μg BUD.

The significantly greater HRQoL improvement with SAL/FP at Week 28, and subsequent loss of significance at Week 52 is particularly important in terms of supporting the clinical relevance of a regular review for patients with persistent asthma. Patients are recommended to undergo regular clinical review and adjust their dose to maintain asthma control every 1–6 months [1], and the loss of significance at Week 52 may, in part, reflect the fact that patients following the stable dosing regimen of SAL/FP were not permitted to step up the dose of regular therapy to gain asthma control.

The recently reported Gaining Optimal Asthma controL (GOAL) study has demonstrated the benefits to patients of an increasing dose of asthma medication. In the GOAL study, additional benefits were achieved with a strategy of aiming for 'Total Control' of asthma – a rigorous composite definition derived from GINA/NIH management guidelines – with increased doses of SAL/FP until Total Control or maximum study dose was reached [13]. This strategy resulted in benefits to all patients, not just those achieving Total Control. It is possible, therefore, that the potential efficacy and HRQoL effects of this treatment may have been underestimated in the present study with a stable dosing regimen.

Importantly, the GOAL study [13] revealed that an additional 8–12% of patients achieved Total Control during sustained treatment. Bateman et al postulated that this may reflect more gradual improvements in airway inflammation in response to prolonged dosing [21,22], which may explain the reduction in exacerbations seen with SAL/FP compared with FOR/BUD in the present study [18]. Previous studies have highlighted the importance of focusing on long-term control of airway inflammation in asthma management [23-26]; determining treatment regimens based on the lowest effective dose that controls symptoms alone does not account for inflammatory changes that can occur in the absence of worsening symptoms and therefore pass unnoticed by patients [27].

Investigating the proportions of change in AQLQ score in this study provides a newer approach to assessing HRQoL, providing deeper insight into the impact of asthma and its treatments on HRQoL. Although the regression analysis was post hoc, the *a priori *intent had been to investigate the proportions of patients improving or deteriorating by categorizing the changes in AQLQ, following the suggestion that this may help with interpreting the importance of HRQoL results at an individual patient level [28]. This method has been used in other studies comparing asthma treatments [29]. In addition, accounting for patients whose HRQoL deteriorates is important for management strategies; treatments need to increase the proportion of patients who improve and also reduce the proportion of patients who deteriorate.

As HRQoL often correlates poorly with objective measures of clinical improvement, it needs to be measured independently [29-31]. It is clear that measures of long-term control of airway inflammation such as exacerbation rate should be a focus of asthma treatment. In contrast, the AQLQ only covers a specific period of time so does not directly capture effects on exacerbations that might have occurred outside of this period. As a result, exacerbation rates in clinical trials may vary between treatment arms, whilst HRQoL can remain relatively consistent [31]. In the present study, the endpoint of AQLQ MID improvement plus no exacerbations provides an alternative measure of treatment success that is directly relevant for clinical decision making. With this analysis, more patients receiving SAL/FP achieved treatment success than those receiving FOR/BUD.

The HRQoL analysis included a total of 82.6% of the ITT population although there were no differences in baseline characteristics between the overall and AQLQ populations. This fall in patient numbers can be explained by the fact that not all of the centers participating in the study administered the AQLQ, mainly because a validated translation is not available in all countries. In addition to this, the number of patients completing the AQLQ at Week 4 and then entering the second treatment phase of the study (Weeks 5–52) is the maximum number of patients who could also have values at Weeks 28 and 52. This is a consequence of the study design, which includes strict continuation criteria at Week 4: patients were only eligible to enter the second treatment phase of the study if, in the previous 7 days, they had no night-time awakenings due to asthma and had not used rescue salbutamol on >2 days. Ineligibility for continuation led to a reduction of approximately 15% in the ITT population [18].

The numbers of patients completing the AQLQ in this study as a proportion of those who had entered the second phase were approximately 58–59% at 28 weeks and 54–55% at 52 weeks. The attrition rate of over one-third of patients across the 52 weeks further highlights the need for appropriate methodological considerations when measuring HRQoL, such as using electronic data capture to retain patients [32], obtaining a wider range of validated translations of the AQLQ, and increasing the sample sizes to prevent loss of power. Indeed, the decrease in completer rate in the present study and resulting loss of power may have contributed to the lack of statistical significance between the treatment groups at Week 52 despite maintenance of numerical difference in scores between treatment groups.

Patient-reported outcomes such as the AQLQ have a clear role to play in clinical trials. This study shows that stable dosing with SAL/FP provided greater HRQoL and efficacy benefits compared with AMD of FOR/BUD.

## Competing interests

DP has no shares in pharmaceutical companies. He has received speaker's honoraria for speaking at sponsored meetings from the following companies marketing respiratory products: 3 M, Altana, AstraZeneca, BI, GSK, MSD, Novartis, Pfizer, Schering-Plough. He has received honoraria for advisory panels with; 3 M, Altana, AstraZeneca, BI, GSK, MSD, Novartis, Pfizer, Schering-Plough. He or his research team have received funding for research projects from: 3 M, Altana, AstraZeneca, BI, GSK, MSD, Novartis, Pfizer, Schering-Plough, Viatris. AW and SY are employed by GSK.

## Authors' contributions

DP participated in the design of the analysis and interpretation of the data, and preparation and revision of the draft article. AW interpreted the data, and revised the article. SY performed the statistical analyses, interpreted the data, and revised the article. All authors read and approved the final manuscript.
